# Structural Insight into the Critical Role of the N-Terminal Region in the Catalytic Activity of Dual-Specificity Phosphatase 26

**DOI:** 10.1371/journal.pone.0162115

**Published:** 2016-09-01

**Authors:** Eun-Young Won, Sang-Ok Lee, Dong-Hwa Lee, Daeyoup Lee, Kwang-Hee Bae, Sang Chul Lee, Seung Jun Kim, Seung-Wook Chi

**Affiliations:** 1 Disease Target Structure Research Center, Korea Research Institute of Bioscience and Biotechnology, Daejeon, Republic of Korea; 2 Metabolic Regulation Research Center, Korea Research Institute of Bioscience and Biotechnology, Daejeon, Republic of Korea; 3 Department of Biological Sciences, Korea Advanced Institute of Science and Technology, Daejeon, Republic of Korea; Universitetet i Bergen, NORWAY

## Abstract

Human dual-specificity phosphatase 26 (DUSP26) is a novel target for anticancer therapy because its dephosphorylation of the p53 tumor suppressor regulates the apoptosis of cancer cells. DUSP26 inhibition results in neuroblastoma cell cytotoxicity through p53-mediated apoptosis. Despite the previous structural studies of DUSP26 catalytic domain (residues 61–211, DUSP26-C), the high-resolution structure of its catalytically active form has not been resolved. In this study, we determined the crystal structure of a catalytically active form of DUSP26 (residues 39–211, DUSP26-N) with an additional N-terminal region at 2.0 Å resolution. Unlike the C-terminal domain-swapped dimeric structure of DUSP26-C, the DUSP26-N (C152S) monomer adopts a fold-back conformation of the C-terminal α8-helix and has an additional α1-helix in the N-terminal region. Consistent with the canonically active conformation of its protein tyrosine phosphate-binding loop (PTP loop) observed in the structure, the phosphatase assay results demonstrated that DUSP26-N has significantly higher catalytic activity than DUSP26-C. Furthermore, size exclusion chromatography-multiangle laser scattering (SEC-MALS) measurements showed that DUSP26-N (C152S) exists as a monomer in solution. Notably, the crystal structure of DUSP26-N (C152S) revealed that the N-terminal region of DUSP26-N (C152S) serves a scaffolding role by positioning the surrounding α7-α8 loop for interaction with the PTP-loop through formation of an extensive hydrogen bond network, which seems to be critical in making the PTP-loop conformation competent for phosphatase activity. Our study provides the first high-resolution structure of a catalytically active form of DUSP26, which will contribute to the structure-based rational design of novel DUSP26-targeting anticancer therapeutics.

## Introduction

Dual-specificity phosphatases (DUSPs) are a group of protein tyrosine phosphatases (PTPs) that can dephosphorylate both phosphoserine/threonine and phosphotyrosine [1]. This group of phosphatases plays a key role in the regulation of cellular signaling cascades, including cell growth, differentiation, and apoptosis [2] by acting on diverse substrates such as phosphoserine, phosphothreonine, phosphotyrosine, inositol phospholipids, and mRNA. Whereas typical DUSPs have a conserved catalytic domain and a mitogen-activated protein kinase (MAPK)-binding (MKB) domain [3], atypical DUSPs include only a catalytic domain, which is similar to those of typical DUSPs, without a substrate-binding domain [1]. Despite their various substrate specificities, DUSPs exhibit a conserved catalytic mechanism mediated by a catalytic triad consisting of a Cys, an Arg, and an Asp in the active site and a phosphate-binding loop (PTP-loop) with a “HCxxGxxRS(T)” consensus motif.

DUSP26 is known to be involved in tumorigenesis and in the progression of cancers. DUSP26 is overexpressed in anaplastic thyroid cancer (ATC) cells and this phosphatase promotes the ATC cell survival by inhibiting the phosphorylation of p38 MAPK, thereby leading to suppression of p38-mediated apoptosis [4]. DUSP26 regulates neuronal cell proliferation [5, 6] and exhibits loss of heterozygosity in breast, prostate, and ovarian cancers [7–9]. It was also shown that Adenylate kinase 2 (AK2) forms a complex with DUSP26 and stimulates the DUSP26 activity, resulting in the dephosphorylation of FADD and the regulation of tumor cell growth [10]. In addition, DUSP26 are found in wild-type p53-containing cancer cells including neuroblastoma, neuroepithelioma, and retinoblastoma cells [11]. In particular, DUSP26 expression is severely unregulated in neuroblastoma cells.

p53 tumor suppressor induces cell cycle arrest, apoptotic cell death, or senescence in response to a variety of stress signals [12–15]. Under the condition of stresses such as DNA damage and hypoxia, the tumor suppression function of p53 is precisely regulated through phosphorylation at many serines and threonines [16–18]. The abrogation of p53 regulation results in tumor progression and defective function of p53 is observed in more than 50% of human cancers [19]. Previous studies also demonstrated that the restoration of wild-type p53 function induced tumor regression *in vivo* through promoting apoptosis and/or senescence [20–22], suggesting that reactivating the p53 function in tumors retaining wild-type p53 is an attractive strategy for anticancer therapy. Recently, it was shown that tumor suppressor p53 is a novel substrate of DUSP26 [23]. DUSP26 dephosphorylates pSer20 and pSer37 in the p53 transactivation domain (TAD) to inhibit the p53 tumor suppressor function and thereby results in the chemoresistance of neuroblastoma cells to doxorubicin-induced apoptosis [23]. DUSP26 inhibition with a small-molecule inhibitor NSC-87877 or shRNA targeting DUSP26 was shown to decrease proliferation and cell viability in neuroblastoma cell lines by activation of p53 and p38 MAPK tumor suppressor pathways [24]. Thus, DUSP26 has been suggested as a novel therapeutic target for the treatment of neuroblastomas that are insensitive to chemotherapy and related pediatric malignancies.

As an atypical DUSP family member, DUSP26 consists of an N-terminal domain (residues 1–60) as well as a conserved catalytic domain (DUSP26-C, residues 61–211). Recently, the crystal structures of wild-type DUSP26-C and mutant DUSP26-C (C152S) were determined [25, 26]. Although they share a similar overall fold with other DUSPs, the DUSP26-C and DUSP26-C (C152S) exhibited the dimeric structures formed by swapping of the C-terminal domain. The structures of DUSP26-C showed that its PTP-loop conformation substantially deviates from the canonical conformation of active DUSPs. Furthermore, a phosphatase activity assay confirmed that DUSP26-C lacks significant catalytic activity [25]. Thus, the structure determination of a catalytically active form of DUSP26 is lacking, and will be necessary to provide a structural template for the rationale design of a novel DUSP26-targeted anticancer drug.

In this study, we determined the crystal structure of a catalytically active form of DUSP26 with an N-terminal extension (residues 39–211, referred to as DUSP26-N) at 2.0 Å resolution. The structure of DUSP26-N (C152S) showed a canonically active conformation of the PTP-loop, which is consistent with the phosphatase activity assay results demonstrating that DUSP26-N exhibited significantly higher catalytic activity than DUSP26-C. Although DUSP26-N (C152S) shares a fundamentally similar structural topology with DUSP26-C, it contains an additional α-helix in the N-terminus, forming the interaction with the α7-α8 loop surrounding the PTP-loop. This interaction stabilizes the canonically active conformation of the PTP loop, suggesting an important role of the N-terminal region in the catalytic activity of DUSP26. Finally, the structure of a catalytically active form of DUSP26 presented here may serve as a useful template for structure-based design of anti-cancer therapeutic agents against human tumors bearing wild-type p53 such as neuroblastoma.

## Results and Discussion

### Phosphatase activity

**Fig 1:**
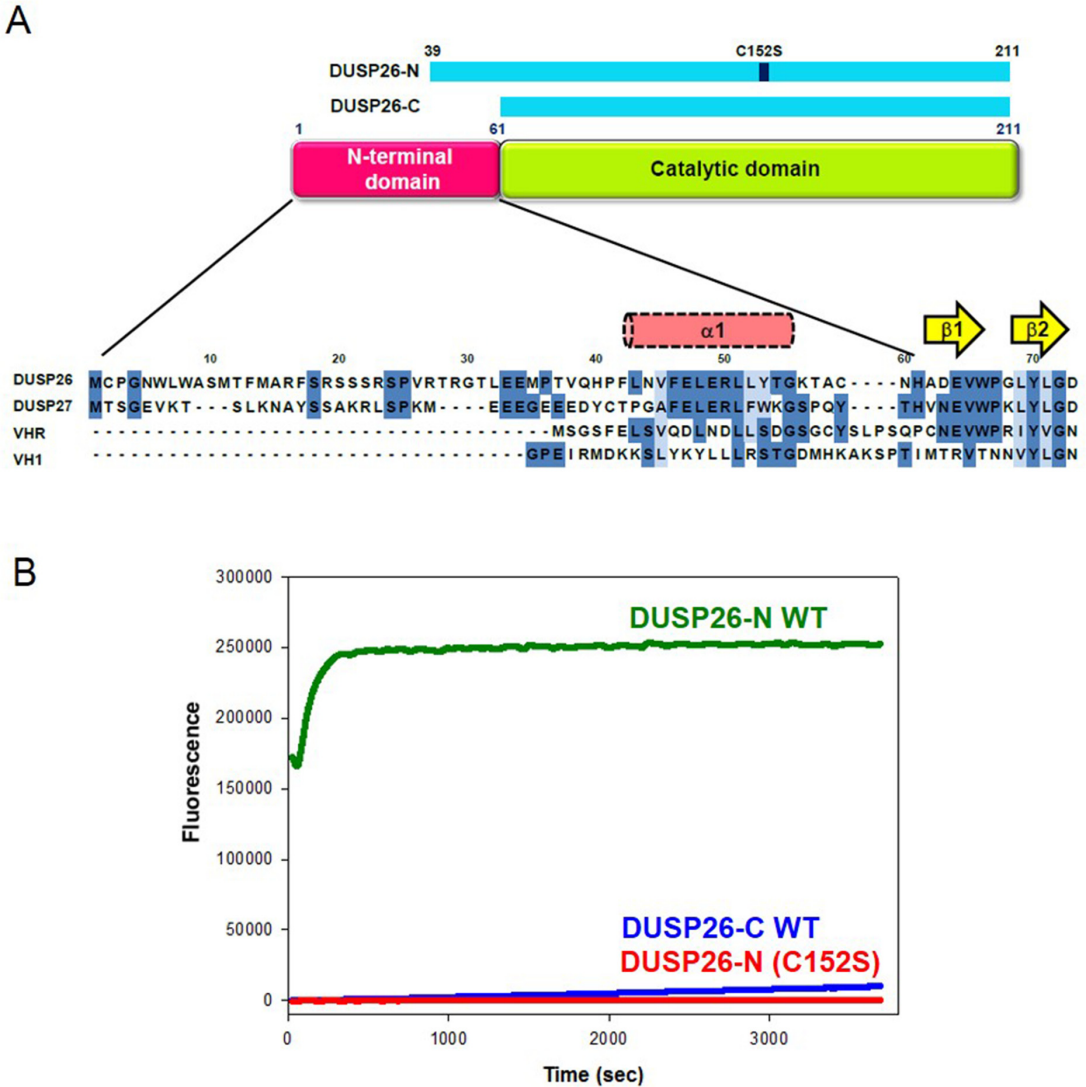
Phosphatase activity of DUSP26-N. (A) Sequence alignment of DUSP26 with DUSP27, VHR, and VH1 in the N-terminal region. The DUSP26-N (C152S) and DUSP26-C (C152S) constructs are shown and the location of the α1-helix in DUSP27 is indicated. The identical residues and homologous residues are colored in dark and light blue, respectively. (B) Phosphatase activity measurements of DUSP26-N using DiFMUP as a substrate. Phosphatase activities of DUSP26-N wild-type (WT) (green), DUSP26-N (C152S) (red), and DUSP26-C WT (blue) were determined by monitoring the fluorescence emitted by the hydrolyzed DiFMU fluorogenic product.

Although two crystal structures of DUSP26-C were previously determined, it showed minimal catalytic activity [25, 26]. Taken together with the presence of an unusually long N-terminal region in DUSP26, the finding of sequence conservation between DUSP26 and DUSP27 in this region (Fig 1A & S1 Fig) prompted us to hypothesize that the N-terminal region in DUSP26 contains an additional α-helix and it might play a role in the catalytic activity. To test this hypothesis, we designed constructs with the additional N-terminal region. Because we were unable to purify full-length DUSP26 because of its insolubility, we used the construct encompassing an extra N-terminal region and catalytic domain (residues 39–211, hereafter referred to as DUSP26-N) for phosphatase activity measurement. We performed a phosphatase activity assay for DUSP26-N using 6,8-difluoro-4-methylumbiliferyl phosphate (DiFMUP) as a substrate. The catalytically inactive C152S mutant form of DUSP26-N (hereafter referred to as DUSP26-N (C152S)), in which the active site cysteine is mutated to serine, was used as a negative control to compare enzyme activity. DUSP26-N exhibited significantly higher phosphatase activity than DUSP26-C at different concentrations, whereas DUSP26-N (C152S) was catalytically inactive against DiFMUP (Fig 1B). In addition, we performed enzyme kinetics analysis with DUSP26-N and compared the kinetic parameters with those of VH1-related DUSP (VHR), DUSP13b, and DUSP14 [27] (Table 1). The K_cat_/K_m_ value of DUSP26-N was 100-fold smaller than that of VHR, but was comparable to that of DUSP13b, which is known as the DUSP that is most analogous to DUSP26 according to phylogenetic analysis [2, 28].

**Table 1:** Kinetic data of DUSP26-N for catalysis of DiFMUP.

Enzyme	Enzyme(n*M*)	*K*_m_ (μ*M*)	*k*_cat_ (min^-1^)	*k*_cat_/*K*_m_ (min^-1^ μ*M* ^-1^)	
DUSP26(39–211)	500	1198.9	12.76	0.11	
VHR	0.2	39.1	408.64	10.45	
DUSP14	1.0	48.5	60.54	1.25	
DUSP13b	25	302.4	147.98	0.49	

### Structure determination and overall structure

**Fig 2:**
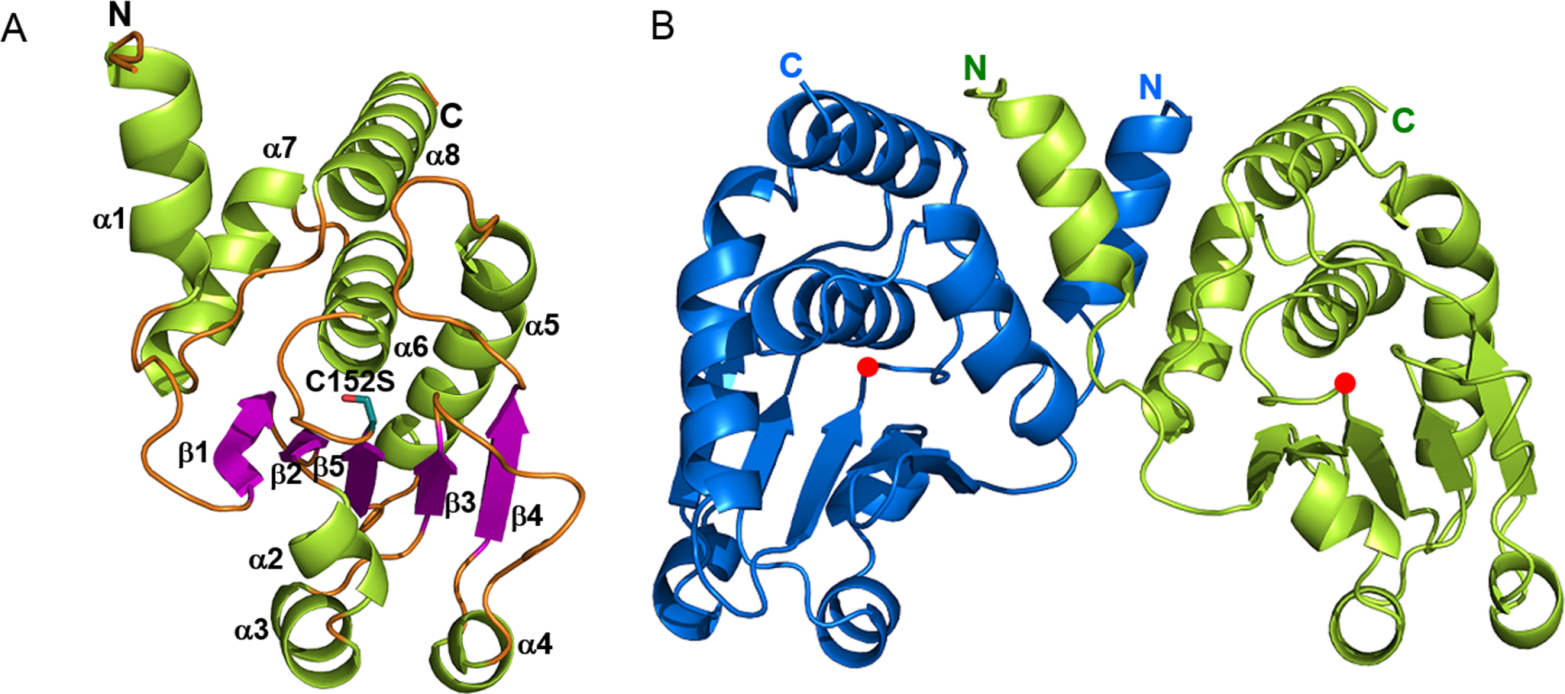
Overall structure of DUSP26-N (C152S). (A) A ribbon model of the monomeric DUSP26-N (C152S) structure. Secondary structural elements are labeled. (B) A ribbon model of the dimeric DUSP26-N (C152S) structure. Chains A and B of DUSP26-N (C152S) are colored blue and green, respectively. The Ser152 residue is shown as a red dot. The N- and C-termini are labeled.

To gain insight into the structural basis for the catalytic activity of DUSP26, we determined the crystal structure of a catalytically active form of DUSP26, DUSP26-N. Because the active site cysteine of the phosphatase with a low p*K*_a_ value is susceptible to oxidation, we mutated the active site cysteine (Cys152) to serine, which does not affect the structure or substrate binding, for crystallization. The crystal structure of DUSP26-N (C152S) was determined and refined to 2.0 Å resolution (Table 2). The structure of DUSP26-N (C152S) was solved in space group C2 by molecular replacement using the DUSP26-C structure as a search model. The overall structural architecture of the DUSP26-N catalytic core domain is fundamentally similar to that of the DUSP26-C monomer. As shown in the structure of DUSP26-C, the DUSP26-N (C152S) monomer contains a central twisted five-stranded β-sheet (β1-β5) sandwiched by two α-helix bundles (Fig 2A). One side of the β-sheet is covered with a three-helix bundle (α2-α4) and the other side is covered with a four-helix bundle (α5-α8). The superimposition of the DUSP26-N (C152S) and DUSP26-C (C152S) structures exhibited a root mean square deviation (RMSD) of 0.37 Å for 121 atoms, excluding the N-terminal α1-helix and C-terminal α8-helix.

**Table 2:** Data-collection and refinement statistics.

Data collection		
	Wavelength (Å)	0.9793
	Space group	C2
	Cell parameter	
	a, b, c (Å)	122.61, 100.80, 70.87
	α, β, γ (°)	90.00, 114.67, 90.00
	Resolution (Å)	50.00–2.00
	Completeness (%)	97.2 (87.7)^b^
	Multiplicity	4.9 (3.1)
	*R*_merge_^a^ (%)	7.4 (30.2)
	<*I*/σ(*I*)>	17.7 (2.6)
Refinement		
	Resolution (Å)	39.69–2.00
	Number of reflections	51,819
	Number of atoms	5,849
	*R*_cryst_ / *R*_free_^c^ (%)	19.8/25.2
RMSD from ideal geometry		
	Bond lengths (Å)	0.007
	Bond angles (°)	0.910
Temperature factors		
	Protein (Å^2^)	31.7
	Main-chain atoms (Å^2^)	30.1
	Side-chain atoms and waters (Å^2^)	33.1
MolProbity		
	Overall score	1.42 (98th percentile)
	Clash score	4.93 (98th percentile)
Ramachandran plot		
	Favored (%)	96.06
	Allowed (%)	3.94
	Disallowed (%)	0.0

^a^ *R*merge = ∑*hk*l ∑*i* |*Ii*(*hkl*)—<I(*hkl*)>|/∑*hkl* ∑*iIi*(*hkl*), where *Ii*(*hkl*) is the intensity of the ith measurement of an equivalent reflection with indices hkl.

^b^ Values in parentheses are for the highest resolution shell.

^c^ The *R*_free_ value was calculated using 5% of the data.

Despite their close resemblance, the most noticeable difference between DUSP26-N (C152S) and DUSP26-C lies in the presence of an additional α1-helix in the N-terminus of DUSP26-N (C152S). This N-terminal α1-helix projects away from the catalytic core in DUSP26-N (C152S) to form a cross-over with the α1′-helix from the other monomer (Fig 2B). Another structural feature of DUSP26-N (C152S) that distinguishes it from DUSP26-C is found in the orientation of a C-terminal α8-helix (residues 192–207). The corresponding C-terminal α7-helix in DUSP26-C projects away from the catalytic core, forming C-terminal domain-swapped dimerization. By contrast, similar to VHR [29], the DUSP26-N (C152S) monomer adopts a “fold-back” conformation of the C-terminal α8-helix, which is packed against the catalytic core from the same subunit to form a four-helix bundle. The packing interaction is mediated through extensive hydrogen bonds of the fold-back α8-helix with its surrounding region such as the β4-α5 loop and α5-helix, including Asn191-Pro122, Asn191-Ser121, Gln197-Phe124, Gln197-Met126, and Gln197-Ser127 hydrogen bonds. Furthermore, the interaction is stabilized by hydrophobic interactions between the α8-helix and α6-helix.

A search for homologous structures using the DALI server [30] identified several members of DUSP family, including DUSP27 (PDB code: 2Y96, z-score = 28.2) [31], DUSP13b (PDB code: 2GWO, z-score = 26.6) [32], and VHR (PDB code: 1VHR, z-score = 25.6) [29]. When the structure of DUSP26-N (C152S) was aligned with those of DUSP27 and VHR except for the N-terminal region, 131 and 129 of the 173 DUSP26-N (C152S) residues could be superimposed with RMSDs of 0.45 Å and 0.59 Å, respectively. In DUSP26-N (C152S), residues 42–207 in chains A and C, residues 41–208 in chain B, and residues 40–207 in chain D were obviously visible in the electron density maps. The quality of the electron-density maps for the active site residues is shown in S2 Fig.

### Active site conformation

**Fig 3:**
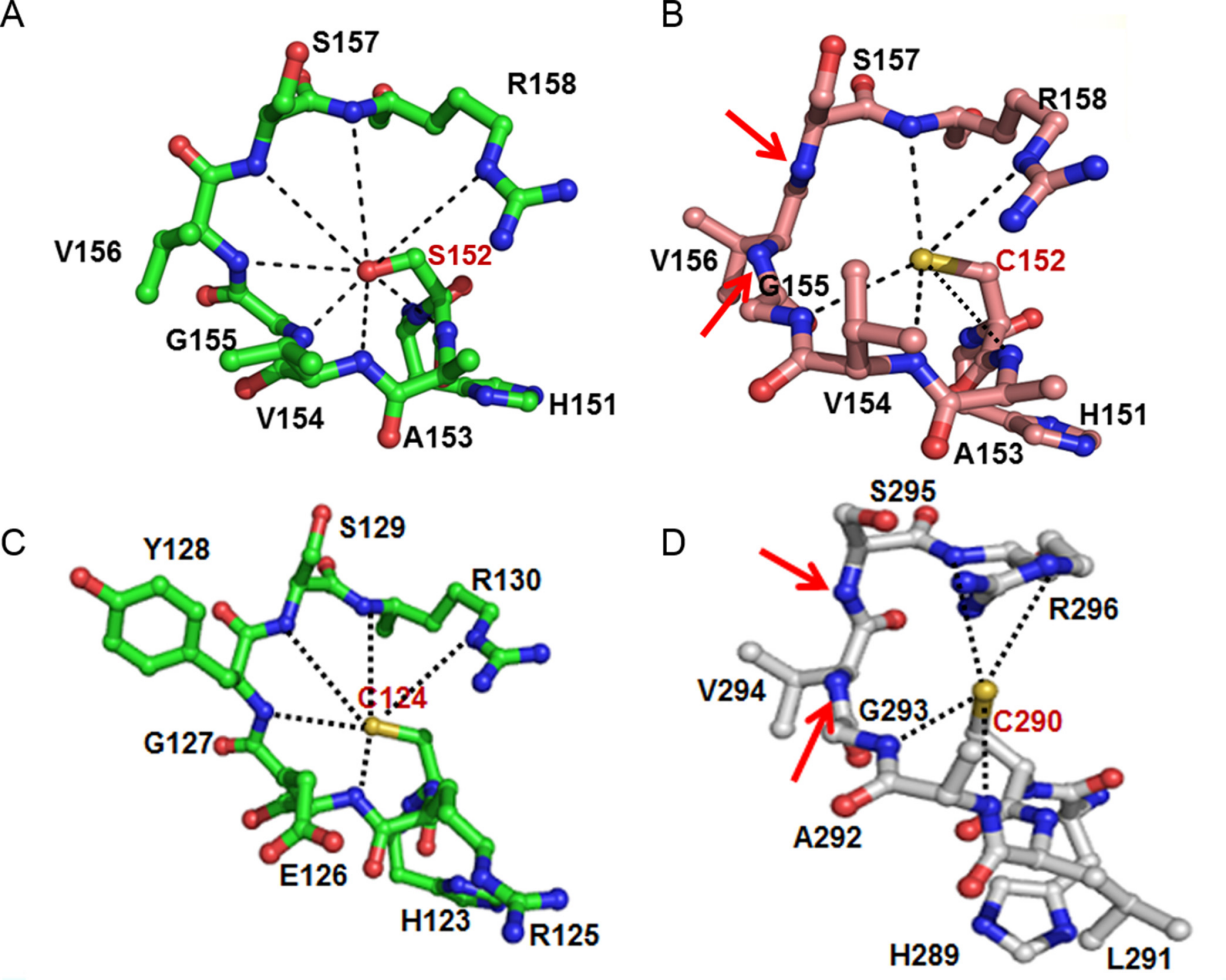
Comparison of the PTP-loop conformation between DUSP26-N (C152S) and other DUSPs. The PTP-loop conformation of DUSP26-N (C152S) (A), DUSP26-C (PDB code: 2E0T) (B), VHR (PDB code: 1VHR) (C), and MKP-4 (PDB code: 3LJ8) (D) are shown. The red arrows highlight the backbone amide atoms that are flipped away from the active site.

As described in detail previously [25], the active site of DUSP26-N (C152S) is composed of the PTP-loop with the “HCxxGxxRS(T)” consensus sequence and a general acid residue. The hydroxyl group of Ser152, corresponding to the catalytic thiol of nucleophilic Cys152, in DUSP26-N (C152S) is surrounded by the PTP-loop, which contains His151-Cys(Ser)-Ala-Val-Gly-Val-Ser-Arg-Ser159 in the loop connecting the β5-strand and the α6-helix. The general acid residue Asp120 in DUSP26-N (C152S) occupies a site corresponding to that of Asp92 in VHR, making a hydrogen bond with the side-chain of Ser157. Structural comparison with the catalytically active VHR and catalytically inactive DUSP26-C and MKP-4 showed that DUSP26-N (C152S) adopts a canonically active PTP-loop conformation, which is highly similar to that of VHR (Fig 3A and 3C). However, the main-chain dihedral angles of Val156 and Ser157 in DUSP26-C (Fig 3B) and of Val294 and Ser295 in MKP-4 (Fig 3D) deviate largely from those of the corresponding residues in DUSP26-N (C152S). Unlike those of DUSP26-C, which are flipped away from the active site, the backbone amide atoms of Val156 and Ser157 in the PTP loop of DUSP26-N (C152S) are pointed into the active site pocket. Together with the backbone amides of residues 153–154 and the guanidinium group of Arg158, they generate a positive electrostatic potential, which is crucial for the catalytic reaction of the cysteine thiolate anion (Fig 3A). The structural superposition of the PTP-loop residues between DUSP26-N (C152S) and VHR showed an RMSD of 0.27 Å, indicating that resultant orientation of PTP-loop in DUSP26-N (C152S) is analogous to that of VHR. Thus, DUSP26-N (C152S) adopts a catalytically active conformation of the PTP-loop, which is very consistent with the phosphatase activity assay results of DUSP26-N.

### Oligomerization status in solution

**Fig 4:**
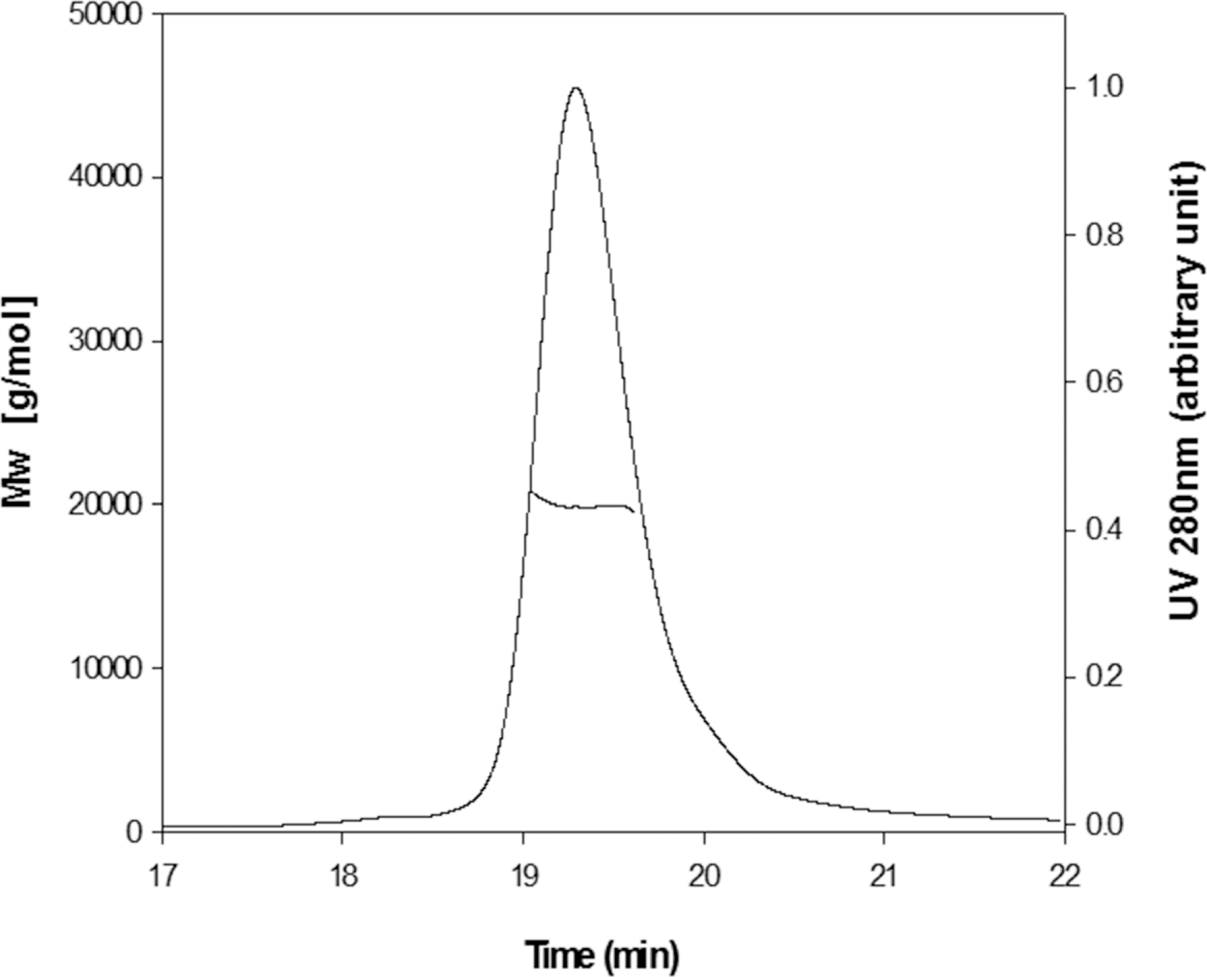
Oligomerization state of DUSP26-N (C152S) in solution as determined by SEC-MALS. The molecular mass of DUSP26-N (C152S) was calculated from the elution profile examined by analytical SEC-MALS. The molecular weight determined by SEC-MALS corresponds to a monomer of DUSP26-N (C152S). The corresponding theoretical mass of DUSP26-N (C152S) is 20,000 Da.

The structures of DUSP27 and Vaccinia virus H1 (VH1) exhibited the N-terminal domain-swapped dimerization [31, 33], whereas the DUSP26-C structure showed the C-terminal domain-swapped dimerization [25]. In the DUSP26-N (C152S) crystal, there are four molecules in the asymmetric unit, giving a V_M_ value of 2.41 Å^3^ Da^-1^ with the solvent content of 49%. DUSP26-N (C152S) forms a dimer with a symmetry-related molecule via an N-terminal cross-over (Fig 2B). The N-terminal α1-helix residues of one protomer forms a dimeric interface with α1'-helix residues from the other protomer. The oligomerization status of DUSP26-N (C152S) in solution was monitored by size exclusion chromatography (SEC) combined with multiangle laser scattering (MALS) detection. The DUSP26-N (C152S) protein eluted as a single peak with a retention time between 19.00 and 19.63 min, which corresponds to the molecular weight of 20,000 Da (estimated MW = 20,647 Da) (Fig 4). Thus, the SEC-MALS data indicate that the DUSP26-N (C152S) protein exists as a monomer in solution. This is consistent with the previous observation that monomeric VHR is catalytically active.

### Structural comparison between DUSP26-N (C152S) and other DUSPs

**Fig 5:**
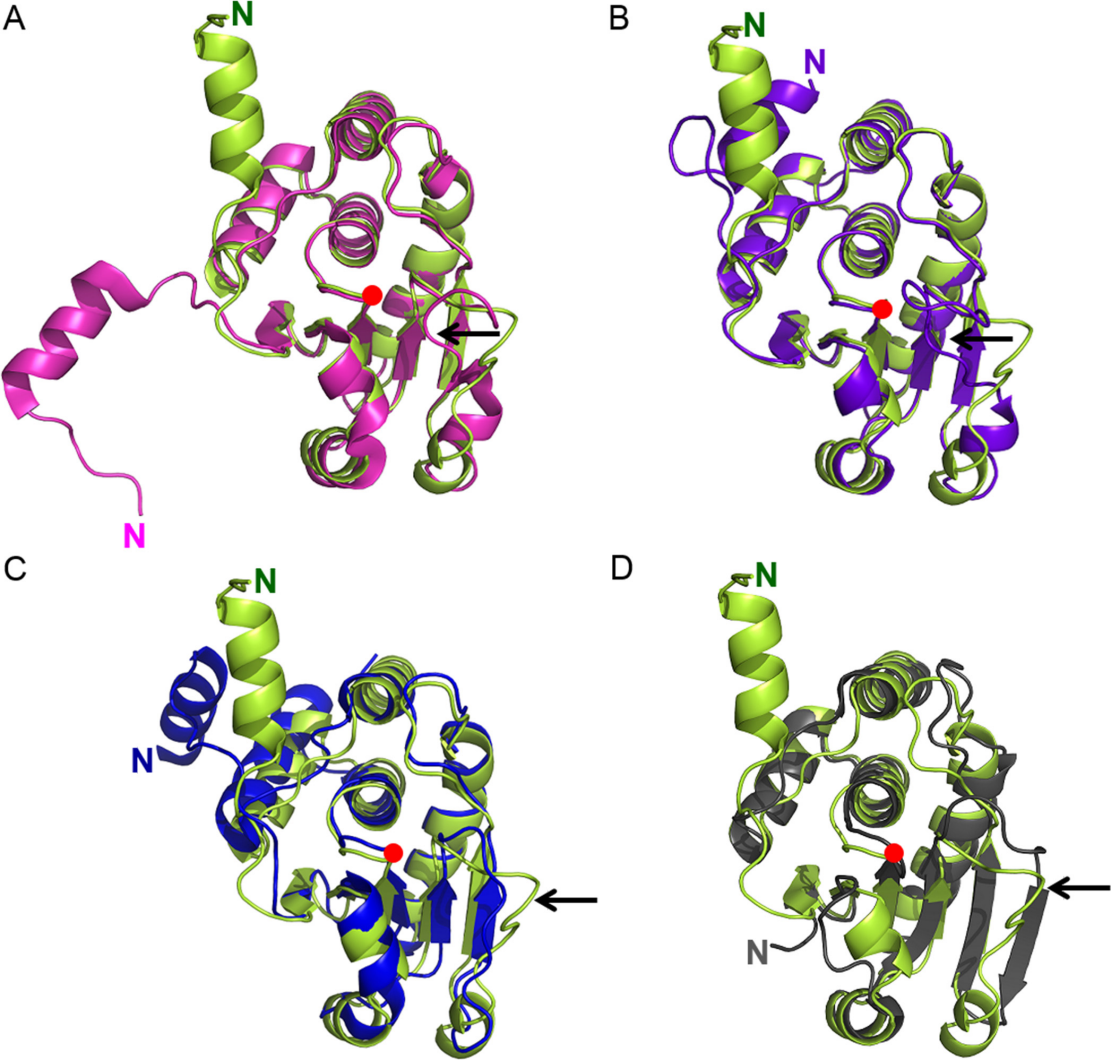
Structural comparison between DUSP26-N (C152S) and other DUSPs. Structural superposition of DUSP27 (magenta) (A), VHR (violet) (B), VH1 (blue) (C), and MKP-4 (gray) (D) onto DUSP26-N (C152S) (green). The large structural difference between the β3-α4 loop of DUSP26-N (C152S) and the corresponding loops of other DUSPs is indicated by black arrows. A red dot indicates the location of the C_α_ atom of the catalytic cysteine.

Despite the high structural convergence among DUSPs, the topology and surface charge distribution of the substrate-binding site are fairly different, which is important for catalytic activity. Comparison of the electrostatic potential of the surfaces showed a significant difference in the electrostatic character and topology of the substrate-binding site between DUSP26-N (C152S) and DUSP26-C (S3 Fig). The positively charged residues are localized near the active site of DUSP26-N (C152S). These residues constitute a substrate-binding pocket of 4.4 Å deep, which a phosphoryl group of substrate can enter. This structural feature of the substrate-binding site is commonly found in other catalytically active DUSPs, such as DUSP27, VHR, and VH1 (Fig 5). In contrast, in the DUSP26-C structure, the entry into the substrate-binding pocket would be closed by the hydrophobic side-chains of Ala153 and Val154 in the PTP-loop (S3 Fig), thereby blocking access of the phosphoryl group in the substrate into the catalytic cysteine at the bottom of the substrate-binding pocket. This is consistent with the finding that DUSP26-N showed greatly higher phosphatase activity than DUSP26-C.

Interestingly, the structural superposition of DUSP26-N (C152S) and other DUSPs revealed a unique conformation of the β3-α4 loop in DUSP26-N (C152S), which is distinct from those of other catalytically active DUSPs such as VHR and DUSP27. The β3-α4 loop of DUSP26-N (C152S) is flipped away from the active site, deviating by a large magnitude of ~10 Å in the C_α_ atom from the corresponding loops of VHR and DUSP27 (Fig 5). Despite the substantial difference in catalytic activity, the β3-α4 loop conformation of DUSP26-N (C152S) is analogous to that of DUSP26-C and the loop occupies essentially the same site as that filled by the β4-strand of catalytically inactive MKP-4 (Fig 5D). Whereas several hydrogen bonds between the PTP-loop and its surrounding β3-α4 loop are found in VHR and DUSP27, the corresponding hydrogen bonds are absent in DUSP26-N (C152S), indicating that the interaction between them is dispensable for the phosphatase activity of DUSP26-N. However, we cannot exclude the possibility that the non-canonical β3-α4 loop conformation of DUSP26-N (C152S) might be involved in substrate specificity. VHR shows higher specificity to phospho-tyrosine, whereas DUSP26 is more specific to phospho-serine and phospho-threonine. The β3-α4 loop of VHR, including the residues Phe68 and Met69, forms one side of the substrate-binding pocket (S3 Fig), which makes the binding pocket narrower and deeper than that of DUSP26-N (C152S). By contrast, the flipped loop conformation away from the binding pocket enables DUSP26-N (C152S) to adopt a different shape of the active site to accommodate cognate substrates.

### Critical role of the N-terminal region in the catalytic activity of DUSP26-N

Typical DUSPs contain an MKB domain in the N-terminus, which acts as a substrate-binding domain. For example, MKP-3 is suggested to exhibit a substrate-induced activation mechanism, where substrate binding to the MKB domain induces activation of the phosphatase [34]. Although it belongs to atypical DUSP, DUSP26 has an unusually long N-terminal region compared to other atypical DUSPs. Our previous finding that DUSP26-C had negligible catalytic activity led us to investigate a functional role of this unusually long N-terminal region in DUSP26. In this study, the phosphatase assay results showed that DUSP26-N has substantially higher phosphatase activity than DUSP26-C, indicating that the addition of N-terminal region encompassing residues 39–60 dramatically enhanced the catalytic activity of DUSP26. This suggests a critical role of the N-terminal region in DUSP26 for its catalytic activity.

**Fig 6:**
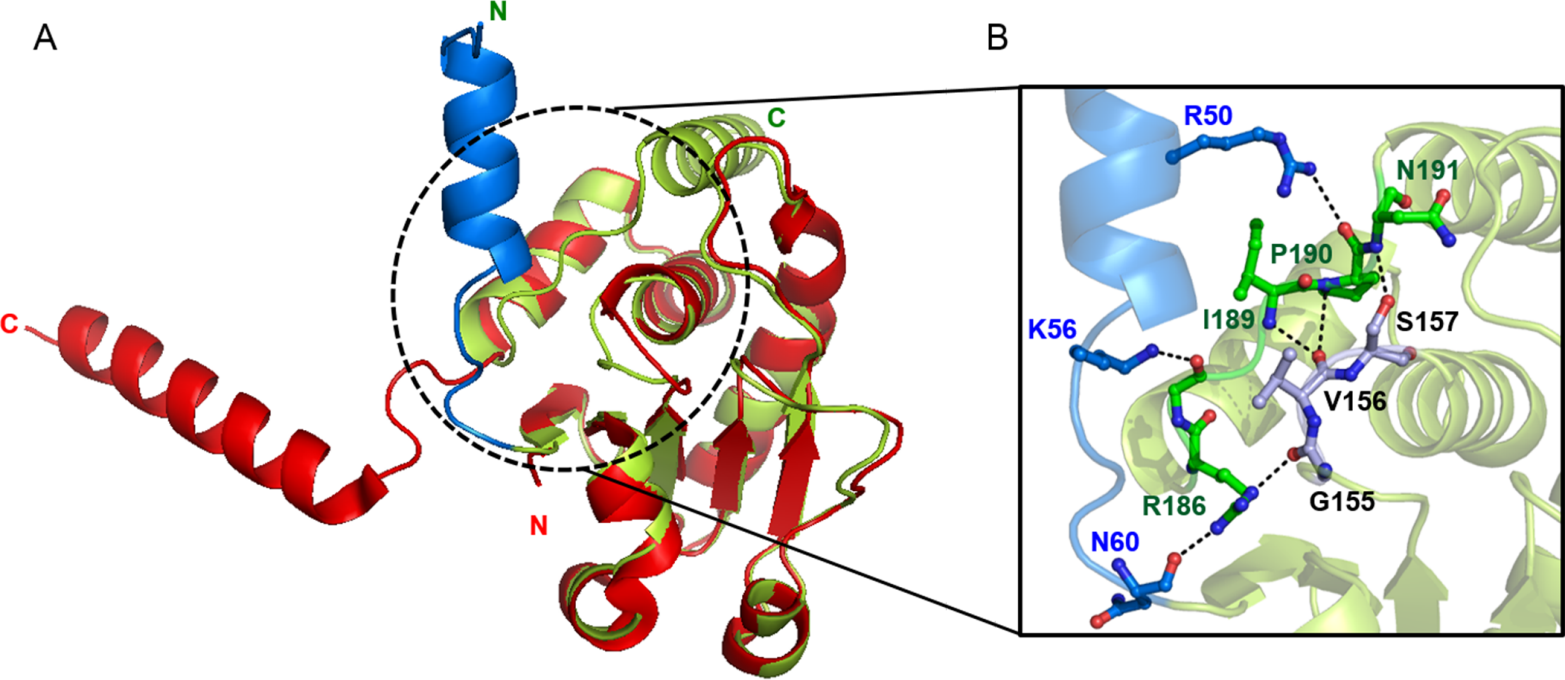
Critical role of the N-terminal region in the catalytic activity of DUSP26-N. (A) Superposition of the DUSP26-C monomer (red) onto the DUSP26-N (C152S) monomer (green). The black dotted circle highlights the large conformational difference in the α7-α8 loop between DUSP26-N (C152S) and DUSP26-C. The additional N-terminal region (residues 39–60) of DUSP26-N (C152S) is colored blue. (B) Hydrogen bonding interaction network among the PTP-loop, its surrounding α7-α8 loop, and α1-helix in DUSP26-N (C152S). Residues in the PTP-loop (Gly155, Val156, and Ser157) and the N-terminal domain (Arg50, Tyr53, and Lys56) form hydrogen bonds with residues in the α7-α8 loop. The residues in the N-terminal domain, α7-α8 loop, and PTP-loop are shown as light blue, green, and dark blue, respectively. Hydrogen bonds are indicated by dotted lines.

Our detailed structural comparison between DUSP26-N (C152S) and DUSP26-C unveiled a structural basis for the critical role of the N-terminal region in the catalytic activity of DUSP26 (Fig 6). Generally, a canonically active PTP-loop conformation of DUSPs is retained by hydrogen bonds between the PTP-loop and its surrounding loops. In the DUSP26-N (C152S) structure, the canonically active PTP-loop conformation is stabilized through formation of hydrogen bonds with the surrounding α7-α8 loop: Gly155-Arg186, Val156-Ile189, Val156-Pro190, and Ser157-Asn191 (Fig 6B). However, because of the absence of this type of interaction between the equivalent surrounding loop and the PTP loop, a significant gap is created in the corresponding site in the DUSP26-C structure, which results in the loss of catalytic activity (Fig 6A). Notably, close inspection of the DUSP26-N (C152S) structure revealed that the additional N-terminal region (residues 39–60) plays a scaffolding role by precisely positioning the surrounding α7-α8 loop for optimal interaction with the PTP loop through formation of hydrogen bonds between the N-terminal residues and the α7-α8 loop: Arg50-Pro190, Tyr53-Gly187, Lys56-Gly187, and Asn60-R186 (Fig 6B). This seems to be critical in stabilizing the canonically active PTP-loop conformation. In addition, the domain-swapping of the C-terminal α8-helix found in DUSP26-C would be blocked by steric hindrance of the additional N-terminal region that is present only in DUSP26-N (C152S) (Fig 6). In the DUSP26-N (C152S) structure, the equivalent C-terminal α8-helix folds back to the active site, which seems to be important for maintaining the PTP-loop in a canonically active configuration.

Although the N-terminal α1-helices are found in a subgroup of atypical DUSPs (from the G4 group in the phylogenetic tree of human DUSPs) [28], there is a noticeable difference in their orientations. In Dusp27 and VH1, the N-terminal α1-helices project away from the catalytic core to mediate dimerization with the other monomer via domain swapping (Fig 5A and 5C) [31, 33]. By contrast, the N-terminal α1-helices in VHR, DUSP13b, and DUSP26-N (C152S) are all similarly located close to the catalytic core (Fig 5B), suggesting their conserved role in the catalytic activities of these DUSPs. Further investigation is needed to determine whether a conformational change of the N-terminal regions in the atypical DUSPs can be utilized for regulation of the catalytic activity, as found in typical DUSPs. Because the functional role of N-terminal residues 1–38 was not determined in this study, this role awaits further studies on the structure of intact full-length DUSP26.

## Conclusions

In summary, we determined the crystal structure of DUSP26-N (C152S) with an additional N-terminal region at 2.0 Å resolution. Although there have been previous structural studies of DUSP26-C reported, the structure of DUSP26-N (C152S) presented here represents the first structure of a catalytically active form of DUSP26. The crystal structure of the DUSP26-N (C152S) showed that it assumes a canonically active PTP-loop conformation, which is consistent with the phosphatase activity assay results that DUSP26-N has significantly higher catalytic activity than that of DUSP26-C. Structural comparison of DUSP26-N (C152S) with the virtually catalytically inactive DUSP26-C revealed that the canonically active PTP-loop conformation is stabilized through formation of an extensive hydrogen bond network between the additional N-terminal region and the α7-α8 loop surrounding the PTP-loop, indicating a critical scaffolding role of the N-terminal region encompassing residues 39–60 for the catalytic activity of DUSP26. Because DUSP26 is a novel therapeutic target for the treatment of neuroblastomas and pediatric malignancies, the crystal structure of DUSP26-N (C152S) will be useful for the rational design of novel DUSP26-targeting anticancer therapeutics.

## Materials and Methods

### Cloning, expression and purification

The wild-type DUSP26-N (residues 39–211) construct was cloned and its catalytically inactive C152S mutant [DUSP26-N (C152S)] was generated as previously described [25]. The regions encoding DUSP26-N and DUSP26-N (C152S) were subcloned into the pET21a vector and expressed in *Escherichia coli* strain BL21(DE3)RIL. DUSP26-N and DUSP26-N (C152S) were expressed and purified as previously reported [25]. The purified proteins were dialyzed against a buffer containing 20 mM HEPES-NaOH (pH 7.0), 50 mM NaCl, and 10 mM dithiothreitol (DTT). Finally, the proteins were concentrated to 10 mg/mL for crystallization.

### Crystallization and data collection

Crystallization of DUSP26-N (C152S) was performed at 18°C using the microbatch methods as previously described [25]. Initial crystallization was performed using commercial screening kits (Hampton Research, Aliso Viejo, CA, USA). DUSP26-N (C152S) crystals were obtained in a drop of 1.5 μL protein solution mixed with 1 μL reservoir solution, which contained 0.1 M Tris-HCl pH 8.5 and 0.9 M potassium thiocyanate. After 5 days, the DUSP26-N (C152S) crystals grew to their full size.

X-ray diffraction data for DUSP26-N (C152S) were collected at up to 2.0 Å resolution on a beamline 7A at the Pohang Accelerator Laboratory (Kyungbuk, Republic of Korea). Diffraction data were processed and scaled using the *MOSFILM* [35] and *SCALA* [36] programs. The DUSP26-N (C152S) crystal belonged to a C2 space group with unit cell parameters of a = 122.61 Å, b = 100.80 Å, c = 70.87 Å, and α = γ = 90.00°, and β = 114.67°.

### Structure solution and refinement

The structure of the DUSP26-N (C152S) was determined by molecular replacement using the DUSP26-C structure as a search model as previously described [25]. Phases were determined using the program *Phaser*. The *CCP4* program [37] placed four molecules of DUSP26-N (C152S) in the asymmetric unit. The interactive manual model was built using the programs *Coot* [38] and *O* [39] and was refined using the program *Phenix* [40]. The structure validation of the final model was assessed using the program *MolProbity* [41]. Statistics for data collection, refinement, and the Ramachandran plot are summarized in Table 1. Figures were drawn using the program *PyMOL* (http://www.pymol.org).

### Phosphatase activity measurements

For the DiFMUP assay, DUSP26-N and the DUSP26-N (C152S) mutant of 3 μM concentration were reacted with 100 μM DiFMUP in a reaction buffer containing 50mM Bis-Tris (pH 6.0) and 1 mM DTT at room temperature (RT) as previously reported [25]. Fluorescence was measured in a fluorescence microplate reader using excitation at 355 nm and emission detection at 460 nm. The steady-state kinetics parameters K_m_ and K_cat_ were determined from a direct fit of the data to the Michaelis-Menten and the Lineweaver-Burk equations.

### SEC-MALS experiments

After system equilibration in 50 mM Bis-Tris (pH 6.0), 1 mM DTT at RT, the samples were analyzed with three detectors in series, namely, the UV and light-scattering detectors of the DAWM HELEOS II system (Wyatt Technology) coupled to a refractive-index detector (Optilab T-rEX refractometer; all from Wyatt Technology Corp., Wyatt Technology Europe GmbH, In der Steubach, Germany). As previously described [42–44], analysis was performed at RT by injecting the DUSP26-N (C152S) protein sample of 100 μL (2.5 mg/mL) into the SEC-MALS system (WTC-015S5 column, Wyatt Technology) at Korea Basic Science Institute in a mobile phase consisting of 50 mM Bis-Tris (pH 6.0) and 1 mM DTT at a flow rate of 0.5 mL/min. Data were analyzed and weight-averaged molar masses were calculated using ASTRA software (V6, Wyatt Technology).

## Accession Numbers

The coordinates and the structural factors of DUSP26-N (C152S) have been deposited in the Protein Data Bank with the accession code 5GTJ.

## Supporting Information

S1 Fig Sequence alignment of DUSP26 with DUSP27 and VHR. The location of secondary structures in DUSP26 is indicated. The identical and homologous residues are aligned in red and blue boxes, respectively.
(TIF)

S2 Fig Stereo image of the electron density map of the active site in DUSP26-N (C152S). The σ_A_-weighted 2*mF*_*o*_*-DF*_*c*_ electron-density map (contoured at the 1.6 σ level) for the active site residues of the PTP-loop of DUSP26-N (C152S). Residues are drawn as sticks with carbon atoms in green, nitrogen atoms in blue, and oxygen atoms in red. Phosphate ion is shown in orange.
(TIF)

S3 Fig Comparison of the electrostatic surface between DUSP26-N (C152S) and other DUSPs. Electrostatic surface representation of the structures of DUSP26-N (C152S) (A), DUSP27 (B), VHR-peptide (DDE(Nle)pTGpYVATR; shown in yellow stick) complex (PDB code: 1J4X) (C), and DUSP26-C monomer (D). Positively charged regions are depicted in blue and negatively charged regions are in red. The green dotted circle indicates the location of the substrate-binding pocket of the DUSPs.
(TIF)

## References

[1] Alonso A, Sasin J, Bottini N, Friedberg I, Osterman A, Godzik A, et al Protein tyrosine phosphatases in the human genome. Cell. 2004;117(6):699–711. Epub 2004/06/10. 10.1016/j.cell.2004.05.018 . 15186772

[2] Patterson KI, Brummer T, O'Brien PM, Daly RJ. Dual-specificity phosphatases: critical regulators with diverse cellular targets. Biochem J. 2009;418(3):475–89. Epub 2009/02/21. . 19228121 10.1042/bj20082234

[3] Farooq A, Zhou MM. Structure and regulation of MAPK phosphatases. Cell Signal. 2004;16(7):769–79. Epub 2004/04/30. 10.1016/j.cellsig.2003.12.008 . 15115656

[4] Yu W, Imoto I, Inoue J, Onda M, Emi M, Inazawa J. A novel amplification target, DUSP26, promotes anaplastic thyroid cancer cell growth by inhibiting p38 MAPK activity. Oncogene. 2007;26(8):1178–87. Epub 2006/08/23. 10.1038/sj.onc.1209899 . 16924234

[5] Wang JY, Yang CH, Yeh CL, Lin CH, Chen YR. NEAP causes down-regulation of EGFR, subsequently induces the suppression of NGF-induced differentiation in PC12 cells. J Neurochem. 2008;107(6):1544–55. Epub 2008/11/19. 10.1111/j.1471-4159.2008.05714.x . 19014381

[6] Wang JY, Lin CH, Yang CH, Tan TH, Chen YR. Biochemical and biological characterization of a neuroendocrine-associated phosphatase. J Neurochem. 2006;98(1):89–101. Epub 2006/06/30. 10.1111/j.1471-4159.2006.03852.x . 16805799

[7] Emmert-Buck MR, Vocke CD, Pozzatti RO, Duray PH, Jennings SB, Florence CD, et al Allelic loss on chromosome 8p12-21 in microdissected prostatic intraepithelial neoplasia. Cancer Res. 1995;55(14):2959–62. Epub 1995/07/15. . 7606709

[8] Pribill I, Speiser P, Leary J, Leodolter S, Hacker NF, Friedlander ML, et al High frequency of allelic imbalance at regions of chromosome arm 8p in ovarian carcinoma. Cancer Genet Cytogenet. 2001;129(1):23–9. Epub 2001/08/25. . 11520561 10.1016/s0165-4608(01)00419-8

[9] Armes JE, Hammet F, de Silva M, Ciciulla J, Ramus SJ, Soo WK, et al Candidate tumor-suppressor genes on chromosome arm 8p in early-onset and high-grade breast cancers. Oncogene. 2004;23(33):5697–702. Epub 2004/06/09. 10.1038/sj.onc.1207740 . 15184884

[10] Kim H, Lee HJ, Oh Y, Choi SG, Hong SH, Kim HJ, et al The DUSP26 phosphatase activator adenylate kinase 2 regulates FADD phosphorylation and cell growth. Nat Commun. 2014;5:3351 Epub 2014/02/20. 10.1038/ncomms4351 24548998 PMC3948464

[11] Tanuma N, Nomura M, Ikeda M, Kasugai I, Tsubaki Y, Takagaki K, et al Protein phosphatase Dusp26 associates with KIF3 motor and promotes N-cadherin-mediated cell-cell adhesion. Oncogene. 2009;28(5):752–61. Epub 2008/12/02. 10.1038/onc.2008.431 . 19043453

[12] Vogelstein B, Lane D, Levine AJ. Surfing the p53 network. Nature. 2000;408(6810):307–10. Epub 2000/12/01. 10.1038/35042675 . 11099028

[13] Harris SL, Levine AJ. The p53 pathway: positive and negative feedback loops. Oncogene. 2005;24(17):2899–908. Epub 2005/04/20. 10.1038/sj.onc.1208615 . 15838523

[14] Vousden KH, Lu X. Live or let die: the cell's response to p53. Nat Rev Cancer. 2002;2(8):594–604. . 12154352 10.1038/nrc864

[15] Chi SW. Structural insights into the transcription-independent apoptotic pathway of p53. BMB Rep. 2014;47(3):167–72. Epub 2014/02/07. 24499665 10.5483/BMBRep.2014.47.3.261 PMC4163879

[16] Kruse JP, Gu W. Modes of p53 regulation. Cell. 2009;137(4):609–22. Epub 2009/05/20. 10.1016/j.cell.2009.04.050 19450511 PMC3737742

[17] Levine AJ. p53, the cellular gatekeeper for growth and division. Cell. 1997;88(3):323–31. Epub 1997/02/07. . 9039259 10.1016/s0092-8674(00)81871-1

[18] Meek DW. Tumour suppression by p53: a role for the DNA damage response? Nat Rev Cancer. 2009;9(10):714–23. Epub 2009/09/05. 10.1038/nrc2716 . 19730431

[19] Hollstein M, Sidransky D, Vogelstein B, Harris CC. p53 mutations in human cancers. Science (New York, NY. 1991;253(5015):49–53. Epub 1991/07/05. . 1905840 10.1126/science.1905840

[20] Ventura A, Kirsch DG, McLaughlin ME, Tuveson DA, Grimm J, Lintault L, et al Restoration of p53 function leads to tumour regression in vivo. Nature. 2007;445(7128):661–5. Epub 2007/01/26. 10.1038/nature05541 . 17251932

[21] Xue W, Zender L, Miething C, Dickins RA, Hernando E, Krizhanovsky V, et al Senescence and tumour clearance is triggered by p53 restoration in murine liver carcinomas. Nature. 2007;445(7128):656–60. Epub 2007/01/26. 10.1038/nature05529 . 17251933 PMC4601097

[22] Martins CP, Brown-Swigart L, Evan GI. Modeling the therapeutic efficacy of p53 restoration in tumors. Cell. 2006;127(7):1323–34. Epub 2006/12/22. 10.1016/j.cell.2006.12.007 . 17182091

[23] Shang X, Vasudevan SA, Yu Y, Ge N, Ludwig AD, Wesson CL, et al Dual-specificity phosphatase 26 is a novel p53 phosphatase and inhibits p53 tumor suppressor functions in human neuroblastoma. Oncogene. 2010;29(35):4938–46. Epub 2010/06/22. 10.1038/onc.2010.244 . 20562916 PMC7580258

[24] Shi Y, Ma IT, Patel RH, Shang X, Chen Z, Zhao Y, et al NSC-87877 inhibits DUSP26 function in neuroblastoma resulting in p53-mediated apoptosis. Cell Death Dis. 2015;6:e1841 Epub 2015/08/08. 10.1038/cddis.2015.207 26247726 PMC4558500

[25] Won EY, Xie Y, Takemoto C, Chen L, Liu ZJ, Wang BC, et al High-resolution crystal structure of the catalytic domain of human dual-specificity phosphatase 26. Acta Crystallogr D Biol Crystallogr. 2013;69(Pt 6):1160–70. Epub 2013/05/23. 10.1107/S0907444913004770 . 23695260

[26] Lokareddy RK, Bhardwaj A, Cingolani G. Atomic structure of dual-specificity phosphatase 26, a novel p53 phosphatase. Biochemistry. 2013;52(5):938–48. Epub 2013/01/10. 10.1021/bi301476m 23298255 PMC3619938

[27] Jeong MS, Kim E, Kang HJ, Choi EJ, Cho AR, Chung SJ, et al A selective Seoul-Fluor-based bioprobe, SfBP, for vaccinia H1-related phosphatase—a dual-specific protein tyrosine phosphatase. Chem Commun (Camb). 2012;48(52):6553–5. Epub 2012/05/25. 10.1039/c2cc31377d . 22622190

[28] Jeong DG, Wei CH, Ku B, Jeon TJ, Chien PN, Kim JK, et al The family-wide structure and function of human dual-specificity protein phosphatases. Acta Crystallogr D Biol Crystallogr. 2014;70(Pt 2):421–35. Epub 2014/02/18. 10.1107/S1399004713029866 . 24531476

[29] Yuvaniyama J, Denu JM, Dixon JE, Saper MA. Crystal structure of the dual specificity protein phosphatase VHR. Science. 1996;272(5266):1328–31. Epub 1996/05/31. . 8650541 10.1126/science.272.5266.1328

[30] Holm L, Sander C. Protein structure comparison by alignment of distance matrices. J Mol Biol. 1993;233(1):123–38. Epub 1993/09/05. 10.1006/jmbi.1993.1489 . 8377180

[31] Lountos GT, Tropea JE, Waugh DS. Structure of human dual-specificity phosphatase 27 at 2.38 A resolution. Acta Crystallogr D Biol Crystallogr. 2011;67(Pt 5):471–9. Epub 2011/05/06. 10.1107/S090744491100970X 21543850 PMC3087626

[32] Kim SJ, Jeong DG, Yoon TS, Son JH, Cho SK, Ryu SE, et al Crystal structure of human TMDP, a testis-specific dual specificity protein phosphatase: implications for substrate specificity. Proteins. 2007;66(1):239–45. Epub 2006/10/18. 10.1002/prot.21197 . 17044055

[33] Koksal AC, Nardozzi JD, Cingolani G. Dimeric quaternary structure of the prototypical dual specificity phosphatase VH1. J Biol Chem. 2009;284(15):10129–37. Epub 2009/02/13. 10.1074/jbc.M808362200 19211553 PMC2665067

[34] Camps M, Nichols A, Gillieron C, Antonsson B, Muda M, Chabert C, et al Catalytic activation of the phosphatase MKP-3 by ERK2 mitogen-activated protein kinase. Science. 1998;280(5367):1262–5. Epub 1998/06/20. . 9596579 10.1126/science.280.5367.1262

[35] Leslie AG. Integration of macromolecular diffraction data. Acta Crystallogr D Biol Crystallogr. 1999;55(Pt 10):1696–702. Epub 1999/10/26. . 10531519 10.1107/s090744499900846x

[36] Evans P. Scaling and assessment of data quality. Acta Crystallogr D Biol Crystallogr. 2006;62(Pt 1):72–82. Epub 2005/12/22. 10.1107/S0907444905036693 . 16369096

[37] Winn MD, Ballard CC, Cowtan KD, Dodson EJ, Emsley P, Evans PR, et al Overview of the CCP4 suite and current developments. Acta Crystallogr D Biol Crystallogr. 2011;67(Pt 4):235–42. Epub 2011/04/05. 10.1107/S0907444910045749 21460441 PMC3069738

[38] Emsley P, Lohkamp B, Scott WG, Cowtan K. Features and development of Coot. Acta Crystallogr D Biol Crystallogr. 2010;66(Pt 4):486–501. Epub 2010/04/13. 10.1107/S0907444910007493 20383002 PMC2852313

[39] Jones TA, Zou JY, Cowan SW, Kjeldgaard M. Improved methods for building protein models in electron density maps and the location of errors in these models. Acta Crystallogr A. 1991;47 (Pt 2):110–9. Epub 1991/03/01. . 2025413 10.1107/s0108767390010224

[40] Adams PD, Afonine PV, Bunkoczi G, Chen VB, Davis IW, Echols N, et al PHENIX: a comprehensive Python-based system for macromolecular structure solution. Acta Crystallogr D Biol Crystallogr. 2010;66(Pt 2):213–21. Epub 2010/02/04. 10.1107/S0907444909052925 20124702 PMC2815670

[41] Chen VB, Arendall WB 3rd, Headd JJ, Keedy DA, Immormino RM, Kapral GJ, et al MolProbity: all-atom structure validation for macromolecular crystallography. Acta Crystallogr D Biol Crystallogr. 2010;66(Pt 1):12–21. Epub 2010/01/09. 10.1107/S0907444909042073 20057044 PMC2803126

[42] Minton AP. Recent applications of light scattering measurement in the biological and biopharmaceutical sciences. Anal Biochem. 2016;501:4–22. Epub 2016/02/21. 10.1016/j.ab.2016.02.007 . 26896682 PMC5804501

[43] Simon AC, Zhou JC, Perera RL, van Deursen F, Evrin C, Ivanova ME, et al A Ctf4 trimer couples the CMG helicase to DNA polymerase alpha in the eukaryotic replisome. Nature. 2014;510(7504):293–7. Epub 2014/05/09. 10.1038/nature13234 24805245 PMC4059944

[44] Peisley A, Wu B, Xu H, Chen ZJ, Hur S. Structural basis for ubiquitin-mediated antiviral signal activation by RIG-I. Nature. 2014;509(7498):110–4. Epub 2014/03/05. 10.1038/nature13140 . 24590070 PMC6136653

