# Supplementary figures and images for: Structural Insight into the Critical Role of the N-Terminal Region in the Catalytic Activity of Dual-Specificity Phosphatase 26
Source: PLoS One. 2016 Sep 1;11(9):e0162115. doi: 10.1371/journal.pone.0162115 (PMC5008780; doi:10.1371/journal.pone.0162115)

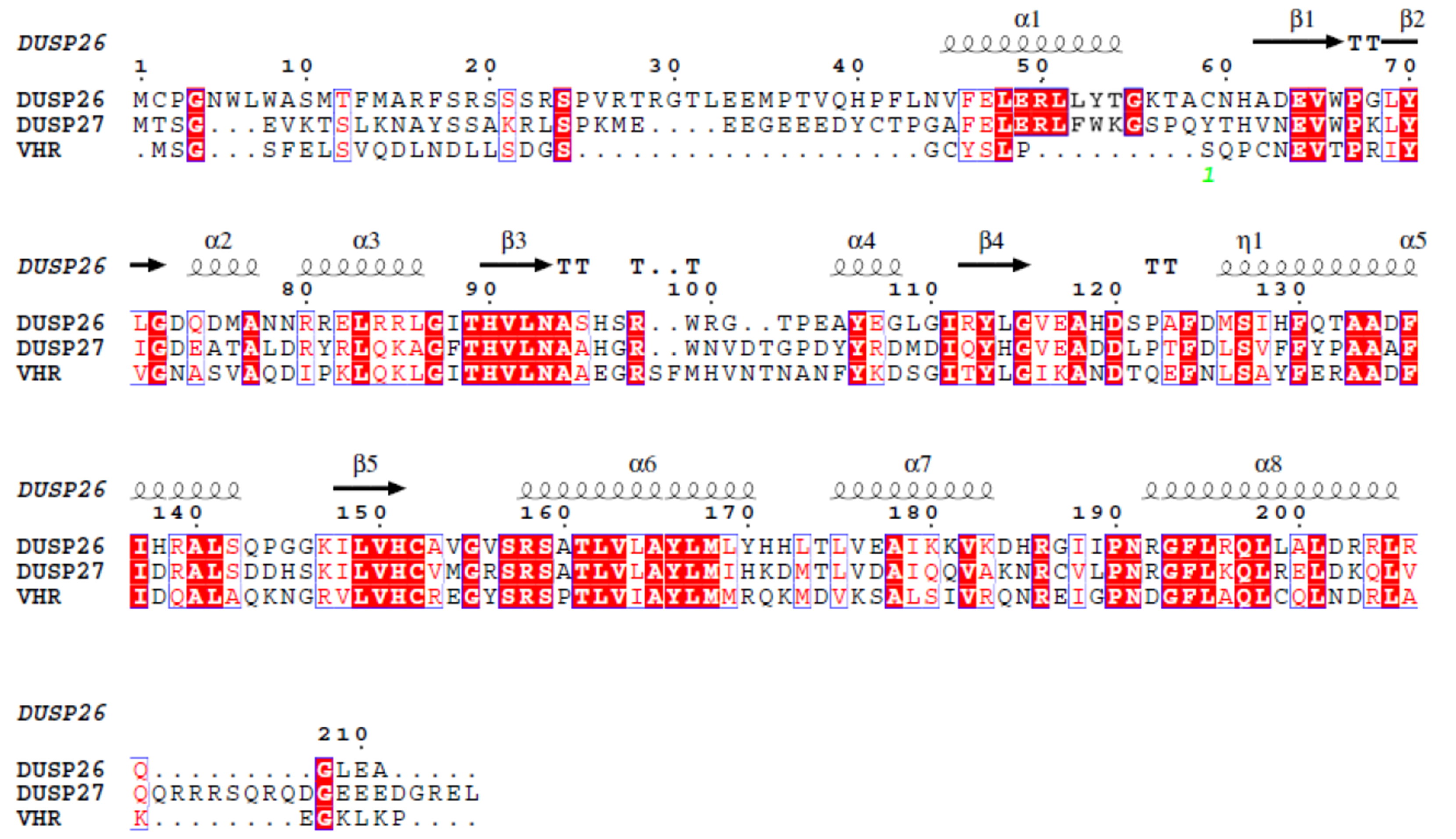

Supplement: S1 Fig — The location of secondary structures in DUSP26 is indicated. The identical and homologous residues are aligned in red and blue boxes, respectively. (TIF) [file pone.0162115.s001.tif]

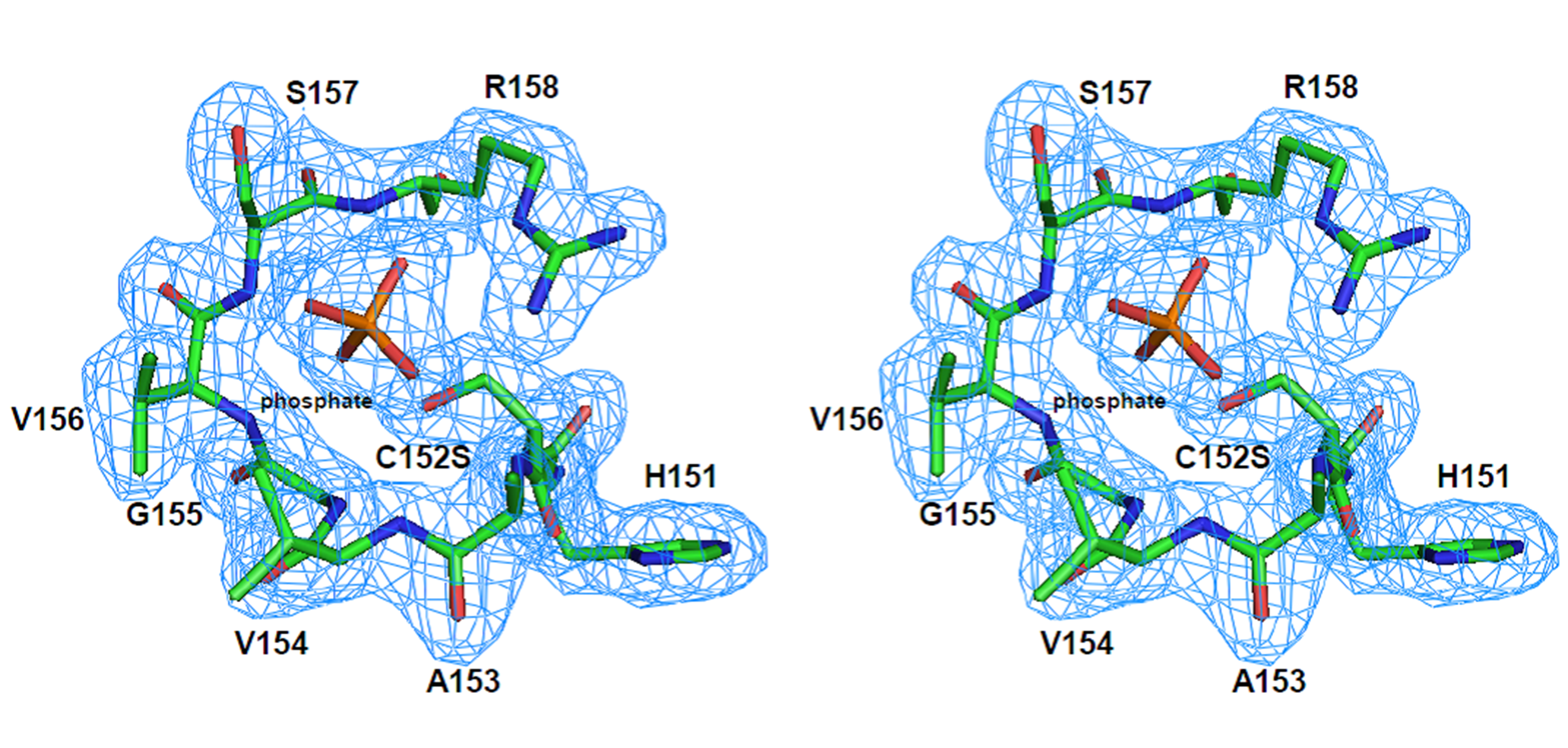

Supplement: S2 Fig — The σA-weighted 2mFo-DFc electron-density map (contoured at the 1.6 σ level) for the active site residues of the PTP-loop of DUSP26-N (C152S). Residues are drawn as sticks with carbon atoms in green, nitrogen atoms in blue, and oxygen atoms in red. Phosphate ion is shown in orange. (TIF) [file pone.0162115.s002.tif]

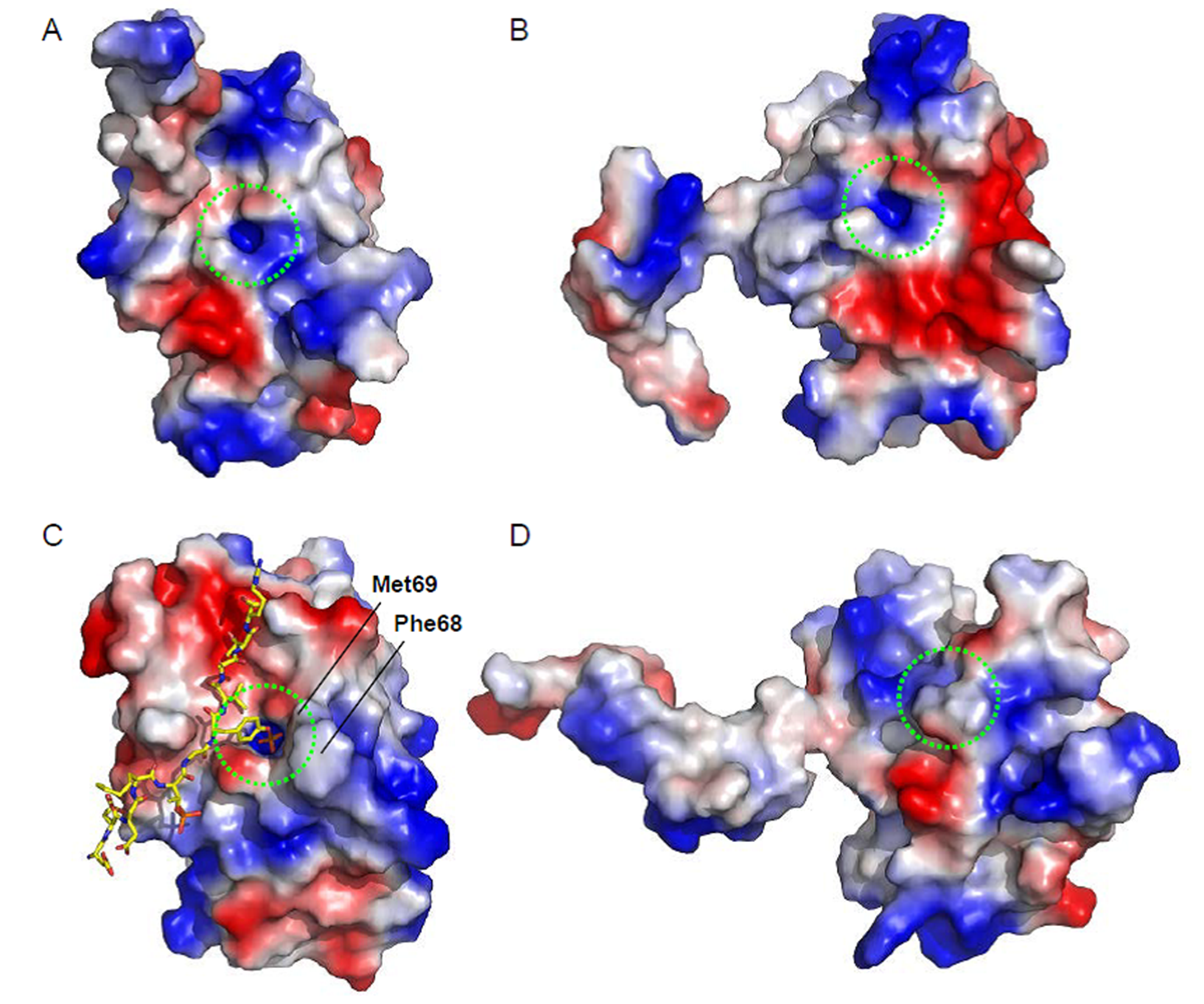

Supplement: S3 Fig — Electrostatic surface representation of the structures of DUSP26-N (C152S) (A), DUSP27 (B), VHR-peptide (DDE(Nle)pTGpYVATR; shown in yellow stick) complex (PDB code: 1J4X) (C), and DUSP26-C monomer (D). Positively charged regions are depicted in blue and negatively charged regions are in red. The green dotted circle indicates the location of the substrate-binding pocket of the DUSPs. (TIF) [file pone.0162115.s003.tif]
